# A novel nonparametric item response theory approach to measuring socioeconomic position: a comparison using household expenditure data from a Vietnam health survey, 2003

**DOI:** 10.1186/1742-7622-11-9

**Published:** 2014-08-12

**Authors:** Daniel D Reidpath, Keivan Ahmadi

**Affiliations:** 1 South East Asia Community Observatory (SEACO), Monash University, Bandar Sunway, Malaysia; 2 Global Public Health, School of Medicine and Health Sciences, Monash University, Bandar Sunway, Malaysia; 3 School of Pharmacy, International Medical University, Kuala Lumpur, Malaysia

**Keywords:** Mokken scale analysis (MSA), Principal component analysis (PCA)

## Abstract

**Background:**

Measures of household socio-economic position (SEP) are widely used in health research. There exist a number of approaches to their measurement, with Principal Components Analysis (PCA) applied to a basket of household assets being one of the most common. PCA, however, carries a number of assumptions about the distribution of the data which may be untenable, and alternative, non-parametric, approaches may be preferred. Mokken scale analysis is a non-parametric, item response theory approach to scale development which appears never to have been applied to household asset data. A Mokken scale can be used to rank order items (measures of wealth) as well as households. Using data on household asset ownership from a national sample of 4,154 consenting households in the World Health Survey from Vietnam, 2003, we construct two measures of household SEP. Seventeen items asking about assets, and utility and infrastructure use were used. Mokken Scaling and PCA were applied to the data. A single item measure of total household expenditure is used as a point of contrast.

**Results:**

An 11 item scale, out of the 17 items, was identified that conformed to the assumptions of a Mokken Scale. All the items in the scale were identified as strong items (H_i_ > .5). Two PCA measures of SEP were developed as a point of contrast. One PCA measure was developed using all 17 available asset items, the other used the reduced set of 11 items identified in the Mokken scale analaysis. The Mokken Scale measure of SEP and the 17 item PCA measure had a very high correlation (r = .98), and they both correlated moderately with total household expenditure: r = .59 and r = .57 respectively. In contrast the 11 item PCA measure correlated moderately with the Mokken scale (r = .68), and weakly with the total household expenditure (r = .18).

**Conclusion:**

The Mokken scale measure of household SEP performed at least as well as PCA, and outperformed the PCA measure developed with the 11 items used in the Mokken scale. Unlike PCA, Mokken scaling carries no assumptions about the underlying shape of the distribution of the data, and can be used simultaneous to order household SEP and items. The approach, however, has not been tested with data from other countries and remains an interesting, but under researched approach.

## Background

Socioeconomic position (SEP) has played an important role in many health studies [1-5]. The relationship between SEP and health has been studied in its own right, [6-8] and it has been treated as a potential covariate/confounder in studies of other substantive causes of poor health [9,10]. Typically, the households in such studies are divided into quintiles according to their estimated SEP and then comparisons are made between fifths of the population [11]. Identifying valid, reliable, acceptable, and low cost methods of measuring SEP is an ongoing and important area of research in the health sciences [11-13].

Among the possible measures of SEP, household expenditure is associated with various health outcomes, [14-16] and tends to be preferred by economists [12]. However, in low and middle income countries expenditure data can be difficult to obtain, [11,13]. and common alternatives have been asset-based indices of household wealth that may include access to utilities and infrastructure. A minority view holds that asset-based measures are in fact superior to expenditure based measures of SEP; [17] with others advocating a middle position. Somi et al., for instance, argued that both expenditure measures and asset-based indices should be treated as legitimate proxies of SEP, given that SEP is a latent variable that cannot be directly observed [18]. In a recent, significant (though not fully comprehensive (p.883)) review of socio-economic measures in low and middle income countries, the authors concluded that the research question, the setting, and the available resources needed to guide the choice of approach to the measurement of SEP [19]. While this is undoubtedly true, in many cases, particularly in the secondary analysis of household survey data there is a tendency to fall back on a small handful of techniques that can be readily applied to data over which the researcher had no control during the collection [20,21].

Various approaches exist for the construction of asset-based indices [13,22]. Ubiquitous among these, which is used in this article as a point of comparison with Mokken scaling, is a principal components analysis (PCA) of a parcel of household assets [17,18,20,21]. The PCA approach was famously, although not first described by Filmer and Pritchett [17] and was adopted by the HNP/Poverty Thematic Group of the World Bank as a standard technique in their poverty and equity analyses covering 44 countries; (cf[23-25]). PCA is the approach taken in the DHS Wealth Index; [26] and it remains a common tool in health research today [7,27,28]. The PCA approach in wealth measurement has its early development in the recognition that multiple measures of wealth create analytic problems associated with collinearity, and PCA offers an efficient data reduction technique to extract orthogonal dimensions [29].

In contrast, Mokken scales take as their conceptual starting point Guttman scales [30,31]. A Guttman scale is a set of ordered (increasingly “harder”) items. Without formally defining “harder” questions, those households with a higher SEP would respond positively to increasingly “harder” questions, leaving a SEP rank order of respondent households from low SEP to high SEP, with all but the highest ranked households eventually finding some questions “too hard” to respond to positively. For example, a question about car ownership is likely to be a “harder” question than a question about ownership of a bucket. Guttman scales, however, are strictly deterministic and do not allow for error in the measurement.

It is here that Mokken scales differ from Guttman scales. Mokken scales are probabilistic, belonging to the non-parametric item response theory model of scales, and allow for stochastic error in measurement [30]. For each item in an asset-based Mokken scale of SEP, the *probability* of a positive response to a question of asset ownership depends on two factors: the SEP latent trait characteristics of the household; and the item characteristics of the asset ownership question. The higher the SEP latent trait of a household, the greater the probability that the household will respond positively to any asset ownership question – without regard to the difficulty of the question itself. The “harder” the ownership question, the lower the probability that a household will respond positively to the question – without regard to the household. This means that a Mokken scale, asset-based index of SEP can rank order households according to their latent trait, and rank order items according to their probability of eliciting a positive response [31]. A Mokken scale analysis (MSA) identifies those items that can be used to rank respondent households according to their probability of a positive response (i.e. their position on the latent trait of SEP), and it orders items according to their probability of being answered positively. Mokken scales are also frequently shorter than scales developed using other procedures, holding out the promise of more concise measures of SEP. While item response theory approaches have been applied to the measurement of household SEP previously, the application has been in high income countries, and relied on parametric techniques (such as the Rasch model) with stronger underlying assumptions than the nonparametric approach of Mokken scales [32-34].

We illustrate the use of MSA in the development of a measure of household SEP in a low income country setting, and contrast Mokken scaling with and equivalent PCA measure of SEP and with a single item measure of household expenditure. The comparison is made using data from a nationally representative household survey conducted in Vietnam. The analysis of a single data set cannot stand as a robust comparison of a scaling technique. It can however illustrate the use of a novel approach to SEP measurement; and in the spirit of an “emerging theme” it may motivate further interest and research.

## Methods

The World Health Survey was a household survey utilising a uniform methodology conducted in 70 countries between 2002 and 2004 [35,36]. Asset ownership questions were included in the survey at the household level. The household asset data analysed here were drawn from the Vietnam, World Health Survey 2003 [37]. Data were collected from 4,154 of 4,174 consenting households (a response rate of 99.5%) [37]. Approximately 23% of households were urban and 77% rural. Households with incomplete asset ownership data (8.7% of households) were excluded from the analysis, leaving a usable sample of 3,810 households (an effective response rate of 91.3%). The level of missing data was considered small enough not to warrant imputation [38]. The survey included 16 dichotomous questions on household asset ownership, access to utilities and infrastructure, and one continuous response question which was dichotomised for the analysis (Table 1).

**Table 1:** The parcel of goods/items used in the Mokken scale analysis

**Item**	**Proportion**^**3**^	**Mokken Scale**	**H**_**i**_^**4**^
Does anyone in your household have a **bicycle**?	83.96	-	-
Does anyone in your household have more than one **house/apartment**^2^	4.67	-	-
How many **cars** are there in your household?^1^	3.65	-	-
Does anyone in your household have a **clock**	90.47	1	.62 (.019)
Does your home have **electricity**?	90.42	1	.56 (.021)
Does anyone in your household have a **television**	78.53	1	.74 (.013)
Does anyone in your household have a **motorbike**	49.79	1	.70 (.013)
Does anyone in your household have a **video/DVD**^2^	31.57	1	.68 (.014)
Does anyone in your household have a **telephone**, fixed-line	16.72	1	.69 (.013)
Does anyone in your household have a **refrigerator**	15.30	1	.69 (.014)
Does anyone in your household have a **magazine subscription**^2^	7.51	1	.46 (.023)
Does anyone in your household have a **washing-machine**	5.33	1	.59 (.021)
Does anyone in your household have a **computer**	5.04	1	.50 (.026)
Does anyone in your household have a **mobile telephone**	4.33	1	.64 (.019)
Does anyone in your household have a **dishwasher**^2^	0.34	1	.57 (.106)
Does anyone in your household have a **bucket**	84.62	2	.49 (.097)
Does anyone in your household have an **agricultural machine**^2^	8.06	2	.49 (.097)

^1^A continuous item dichotomised as none (0) and one or more (1).

^2^These are “Country Specific” items, which vary from country to country in which the World Health Survey was conducted.

^3^The proportion of households owning each asset.

^4^Standard errors are shown in parentheses.

The final items used in both the Mokken scale analysis and the 11 item PCA are identified as belonging to Mokken Scale 1.

Household expenditure was measured with the question: “In the last 4weeks, how much did your household spend in total?”

The data from the World Health Survey are publicly available for analysis as anonymised, unit record files. Ethics Committee approval for the analysis presented here was neither sought nor required.

### Data analysis

PCA is a well described technique for developing asset-based indices of SEP and will not be described in detail here [11]. It is nonetheless worth noting that PCA is a statistical technique to reduce the dimensionality of data by identifying sets of weighted linear combinations (principal components) of the original asset measures, such that each new principal component accounts for a smaller proportion of the variance than the preceding components, and that each of the identified principal components are orthogonal [39]. It is the first principal component (accounting for the greatest proportion of the variance) that is typically used to construct an index of household SEP. An underlying assumption of PCA is that the data are continuous and drawn from a multivariate normal distribution [20]. This is not the case with a series of dichotomous asset-ownership questions. However, assuming an underlying continuous, normally distributed latent variable, the polychoric correlation between two observed dichotomous variables can be used as an estimate of the actual correlation [20]. It was the polychoric correlation matrix that was used in the PCA described here.

MSA relies on an automated procedure for the selection of items that belong to one or more independent Mokken scales (or no scale at all), and a series of methods to investigate the extent to which the scales maintain the assumptions of a nonparametric item response theory model [40].

A Mokken scale of household SEP is based on four assumptions:

1. *Unidimensionality*. A scale of responses to questions of household asset ownership measures a dominant, single latent trait of household SEP.

2. *Local Independence*. Responses to an asset ownership question are not influenced by the responses to any other asset ownership question in the same scale.

3. *Monotonicity*. The probability of a positive response to an asset ownership question is a monotonically increasing function of the latent trait. This assumption would be violated, for instance, if both low and high SEP households had a low probability of owning asset ***a***_***i***_, but middle SEP households had a high probability of owning asset ***a***_***i***_.

4. *Non-intersection*. If the probability of households owning asset ***a***_***i***_ is lower than probability of households owning asset ***a***_***k***_, for one level of the SEP latent trait (e.g., a low SEP household), then it will be lower for all levels of the latent trait (i.e., middle and high SEP households). This is referred to in the literature as *invariant item ordering* or (IIO), and means that the ordering of difficulty of the asset ownership question holds for all households without regard to their SEP [41-43].

In Mokken scaling, the *model of monotone homogeneity* (MMH) is based on the first three assumptions. In its practical application, a household SEP scale meeting the requirements of MMH allows for the ordering of households by the sum of the number of positive responses to the asset ownership questions. The more positive responses, the higher a household’s SEP [41,42].

If a scale meets the requirements of the MMH and the scale meets the fourth assumption of non-intersection (or IIO), it also fulfills the requirements of the *double monotonicity model* (DMM). In its practical application it means that not only can households be ordered on the latent trait of household SEP, but the asset ownership questions (items) can be ordered according to how “hard” or “difficult” they are.

There are various methods for testing the assumptions of a Mokken scale. At the heart of the procedures are Loevinger’s homogeneity coefficients [44,45]. These are three related coefficients, which are used to select items that contribute to a unidimensional (homogeneous) scale [30,44]. For details, readers are referred to a number of articles and books written on the topic, where for brevity we focus on the conceptual and applied application.[40,43,46,47]. The main coefficients used are H_i_ and H. The H_i_ coefficient provides a measure of the scalability of each item *i* that makes up the potential scale, and the H coefficient provides a measure of the scalability of the whole scale (i.e., the degree to which the items always appear in the same relative order ) [48]. Guidelines for the interpretation of the coefficients suggest that values of .3 –.4 are indicative of a weak scale (or item), values of .4–.5 are indicative of a medium scale (or item) and values > .5 are indicative of a strong scale (or item) [30]. When the H coefficient is calculated on the transpose matrix of dichotomous asset ownership responses (H^T^), one obtains a summary statistic of the accuracy of asset ordering within a scale.

The automated item selection procedure (AISP) partitions a set of items into zero or more Mokken scales and provides summary statistics for the items and scales [46]. This is a necessary but not sufficient procedure for establishing a Mokken scale. For the MMH one needs to establish monotonicity, and for the DMM one also needs to establish the non-intersection assumption, or IIO. There are a number of possible approaches to establishing non-intersection, and in this study, the *restscore method* was used, which compares all possible item pairs to establish whether significant violations of the non-intersection assumption occur [43,46].

All analyses were conducted in the R statistical environment [49]. The analyses were supported by the mokken package for the Mokken scale analysis, [47] and the polycor package for estimating polychoric correlations [50].

## Results

### Mokken Scale Analysis

Of the 17 items included in the MSA, the automated item selection procedure identified three items which could not be scaled, 12 items potentially belonged to one scale, and two items potentially belonging to a second scale (Table 1).

For the remainder of the paper, the focus is on the 12 items contributing to scale one. The single item scalability coefficients, H_i_ ranged in value from .46 to .79 with 10 of the 12 items having values greater than 0.5; i.e., potentially “strong” items [30]. The standard errors of each H_i_ were relatively small, with the exception of the item measuring dishwasher ownership. Indeed, of the 10 items with H_i_’s indicating strong scalability, only the H_i_ of the item measuring dishwasher ownership had a 95% confidence interval to include a value less than .5 (i.e., *H*_*i*_ - 1.96 × *SE* = .480) [47,51]. Importantly, there was no item for which the lower bound of the 95% confidence interval included .3, a conventionally used cut-off to reject potential items as un-scalable [51].

**Figure 1:**
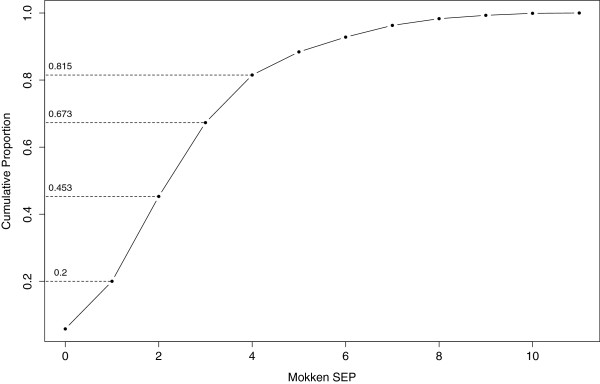
An ogive of the distribution of Mokken SEP scores with approximate quintile boundaries.

The overall scalability coefficient for scale one, H was .65. There were, furthermore, no violations of monotonicity. However the *rest scores*, used to test the IIO, indicated two critical violations associated with electricity availability and clock ownership. It is recommended that the worst offending item is removed from the preliminary scale and the rest scores re-examined – in this case the item related to electricity availability. Once electricity availability had been removed as an item in the scale, there were no further violations [46].In keeping with the Mokken scaling approach, the Mokken SEP score was calculated as the unweighted sum of the 11 remaining dichotomous items. Figure 1 shows the distribution of Mokken SEP scores with approximate quintile boundaries. Twenty percent of households had a score of 1 or less, 45% of households had a score of 2 or less, 67% of households had a score of 3 or less, and 82% of households had a score of 4 or less. The households in the top 20% had scores of 5 or more.

### Principal components analysis

Two separate PCAs were conducted using different item pools. The first PCA was based on the 17 item asset ownership questions from the World Health Survey. The second PCA was based on the reduced, 11 item pool, identified in the Mokken analysis.

In the first analysis of all 17 items, the first principal component accounted for 51.5% of the variance, the second accounted for 20.8% of the variance, and the third accounted for 9.7% of the variance. After the fourth component, eigen values fell below 1. A scree plot (un-shown) indicated no obvious discontinuity or ‘elbow’ in the declining eigen values. In the second PCA, using the 11-item pool, the first principal component accounted for 24.9% of the variance, the second accounted for 21.6% of the variance, and the third accounted for 14.4% of the variance. After the fourth component, eigen values again fell below 1. The scree plot (un-shown) indicated no obvious discontinuity or ‘elbow’ in the declining eigen values.

For both PCA analyses, asset scores based on the first principal component were used to create a continuous SEP score, and quintiles.

The Pearson’s product moment correlation between the Mokken measure and the 17 item PCA measure of SEP was very high, r = .98, and for the quintiles of wealth, Spearman’s rank order correlation was r = .96. The relationship, however, was weaker for the reduced, 11 item PCA measure. The Pearson’s product moment correlation between the Mokken-based measure and the PCA-based measure of SEP was moderate, r = .68, and the Spearman’s rank order correlation for quintiles of wealth was very low r = .11.

### Reliability

Cronbach’s alpha was used as a measure of the reliability three SEP scales. The items were weighted prior to the calculation of Cronbach’s alpha. They were weighted to ensure that each SEP scale was evaluated based on its adjusted item scores.

In the case of the Mokken scale, the items were unit weighted, because the scale is a simple sum of the asset-based items. In the case of the PCA scales, the items were weighted by the PCA loadings from the first principal component. Cronbach’s alpha for the unit weighted 17 item scale was included as a point of contrast.

The reliability of the unit weighted, 17 item scale was .73 (95% CI: .71 – .74). The reliability of the PCA weighted, 17 item scale was .52 (95% CI: .49 – .54). The reliability of the unit weighted, 11 item Mokken scale was .76 (95% CI: .75 – .78). The reliability of the PCA weighted, 11 item scale was .19 (95% CI: .15 – .23). The Mokken scale had a significantly larger Cronbach’s alpha than the other potential SEP scales.

### Comparisons with household expenditure

**Figure 2:**
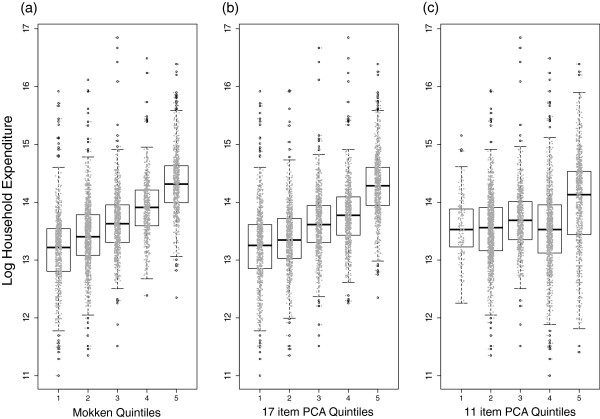
Boxplots of the distribution of log transformed total household expenditure across quintiles of the Mokken SEP (a), a 17 item based PCA SEP (b), and an 11 item based PCA SEP (c).

The responses to the single household expenditure question from the World Health Survey were log transformed because of the long tail of the distribution that is typical of expenditure and income data. Figure 2 shows a box plot of household expenditure data over the quintiles of household SEP estimated by the Mokken scale analysis (2a), the PCA using all 17-items (2b), and the PCA using the 11 items identified by the Mokken analysis (2c). The distribution of actual household expenditure values in each quintile was overlaid as grey coloured points.A visual inspection suggests, as might be expected from the close to perfect correlation between the the 17 item PCA quintiles and Mokken scale quintiles, that they perform very similarly (Figure 2a and b). In contrast the boxplot of the 11 item PCA quintiles against household expenditure shows no obvious systematic relationship (Figure 2c).

The Pearson’s product moment correlation between the household expenditure data and the Mokken SEP continuous scores was r = .59, for the 17 item PCA SEP continuous scores it was r = .57, and for the 11 item PCA SEP continuous scores it was r = .41. The correlations between the household expenditure and the quintiles data for the Mokken (r = .57) and the 17 item PCA SEP (r = .53) showed similar levels of performance. The correlation between the household expenditure data and the 11 item PCA SEP quintiles was r = .18.

## Discussion

Mokken scaling appears to be a promising approach to the development of an asset-based measure of household SEP. The Mokken scale was strongly correlated with the 17 item PCA measure of SEP, and with a significantly better Cronbach’s alpha. Both measures performed similarly with respect to the measure of total household expenditure. That measures of asset based SEP and expenditure were moderately correlated supports the general notion of an underlying latent wealth construct,[12,18] and it supports the use, of asset based measures if expenditure measures are desirable but unobtainable.

In sharp contrast to the 17 item PCA, the correlation was much weaker between the 11 item Mokken scale and the 11 item PCA measure of SEP. Furthermore, the 11 item PCA SEP quintiles showed no practical relationship with the household expenditure; and the Cronbach’s alpha for the PCA measure was very weak.

A novel feature of Mokken scaling is that it orders items as well as households. Outside the direct value of SEP to health research, it may potentially be used to track changes in items indicative of wealth over time. Hard items today, (i.e., items to which only the wealthiest have a high probability of responding positively) may become easy items in the future, or visa-versa. In 2003 when the World Health Survey data for Vietnam was collected, the market penetration of the mobile telephone was less than 5%, supporting the apparent “hardness” of the item identified in the Mokken scale analysis (Table 1). Mobile phones were a rare and expensive commodity in 2003. In 2012 the market penetration of the mobile phone in Vietnam had exceeded 100%, making it an “easy” item that would not today readily separate the wealth quintiles [52]. The mobile phone as an asset item was highlighted for very similar reasons in a recent Rasch analysis of poverty in Europe (p.69) [32].

Given the growth of interest in item response theory approaches to modelling SEP, [33,34]. the results of the Mokken scaling presented here should pique some interest. Unlike parametric item response theory models, there are fewer assumptions associated with Mokken scaling which can broaden its application. This has been found in other areas of health research where Mokken scaling has been used successfully in its own right, and used to support or check parametric item response theory approaches [53-55].

### Limitations

One of the limitations of this analysis relates to the generalisability of the approach; specifically whether Mokken scaling will always perform comparably well or out perform PCA; and whether it will perform as well as other approaches [56]. This limitation, however, needs to be placed in the context of at least one of the paper’s goals, which was to illustrate the use of Mokken scale analysis in the context of SEP measurement.

For some the apparent complexity of Mokken scaling over PCA maybe seen as a limitation; indeed, one of the Reviewers of a draft raised this very possibility. We would argue that the apparent complexity is a function of familiarity. Understanding PCA and the underlying eigen values is not trivial. Exposure to a technique creates familiarity. This is the first paper we know of that uses Mokken scaling in the development of an SEP measure. Furthermore, given the emergence of item response theory approaches in SEP measurement, this is well timed and should add another technique to the quiver of methods available to epidemiologists [56].

The use of a single global measure of household expenditure as a comparative measure of SEP is also a limitation. While additional questions could undoubtedly have improved the measure of household expenditure, these were not available, and as so often happens in secondary data analysis, one is constrained by the choices made by the original researchers. It is also known that expenditure data in low income settings can be hard to obtain, which motivated the creation of asset-based measures in the first place. The real problem with single question measures is that they are often very noisy (in a stochastic sense). The fact that both the 17 item PCA measure of SEP and the Mokken scale measure of SEP correlated moderately with the single item measure of household expenditure, however, suggests that the choice was not misguided in this context.

## Conclusion

The Mokken scale measure of household SEP performed at least as well as PCA, and outperformed the PCA measure developed with the 11 items used in the Mokken scale. Unlike PCA, Mokken scaling carries no assumptions about the underlying shape of the distribution of the data, and can be used simultaneous to order household SEP and item difficulty. The approach, however, has not been tested with data from other countries and remains an interesting, but under researched approach.

## Competing interests

The authors declare that they have no competing interests.

## Authors’ contributions

DDR and KA jointly developed the idea of applying Mokken scaling to the problem of measuring SEP. KA provided technical input on Mokken scaling, DDR conducted the preliminary analysis. DDR and KA jointly drafted and edited the manuscript. All authors read and approved the final manuscript.
